# Tumor necrosis factor-α enhances voltage-gated Na^+^ currents in primary culture of mouse cortical neurons

**DOI:** 10.1186/s12974-015-0349-x

**Published:** 2015-06-26

**Authors:** Weiqiang Chen, Jiangtao Sheng, Jingfang Guo, Fenfei Gao, Xiangfeng Zhao, Jianping Dai, Gefei Wang, Kangsheng Li

**Affiliations:** Department of Microbiology and Immunology, Key Immunopathology Laboratory of Guangdong Province, Shantou University Medical College, 22 Xinling Road, Shantou, 515041, Guangdong China; Department of Neurosurgery, First Affiliated Hospital, Shantou University Medical College, 57 Changping Road, Shantou, 515041, Guangdong China; Department of Pharmacology, Shantou University Medical College, 22 Xinling Road, Shantou, 515041, Guangdong China

**Keywords:** Tumor necrosis factor-alpha, Voltage-gated sodium channels, Cortical neurons, Patch-clamp recording, Neuronal excitability

## Abstract

**Background:**

Previous studies showed that TNF-α could activate voltage-gated Na^+^ channels (VGSCs) in the peripheral nervous system (PNS). Since TNF-α is implicated in many central nervous system (CNS) diseases, we examined potential effects of TNF-α on VGSCs in the CNS.

**Methods:**

Effects of TNF-α (1–1000 pg/mL, for 4–48 h) on VGSC currents were examined using whole-cell voltage clamp and current clamp techniques in primary culture of mouse cortical neurons. Expression of Nav1.1, Nav1.2, Nav1.3, and Nav1.6 were examined at both the mRNA and protein levels, prior to and after TNF-α exposure.

**Results:**

TNF-α increased Na^+^ currents by accelerating the activation of VGSCs. The threshold for action potential (AP) was decreased and firing rate were increased. VGSCs were up-regulated at both the mRNA and protein levels. The observed effects of TNF-α on Na^+^ currents were inhibited by pre-incubation with the NF-κB inhibitor BAY 11–7082 (1 μM) or the p38 mitogen-activated protein kinases (MAPK) inhibitor SB203580 (1 μM).

**Conclusions:**

TNF-α increases Na^+^ currents by accelerating the channel activation as well as increasing the expression of VGSCs in a mechanism dependent upon NF-κB and p38 MAPK signal pathways in CNS neurons.

## Introduction

Tumor necrosis factor-alpha (TNF-α) is a prototypic pro-inflammatory cytokine of the innate immune system [1]. Upon binding to TNFR1 or TNFR2 in the brain, TNF-α modulates a variety of fundamental physiological processes of the central nervous system (CNS), including synapse formation and regulation, neurogenesis, and regeneration [2, 3]. TNF-α is also implicated in many pathological processes and diseases of the CNS, including gliosis, demyelination, blood–brain-barrier deterioration, and cell death [3].

TNF-α inhibits sustained K^+^ channel currents in rat small-diameter sensory neurons [4] and Ca^2+^ channel currents in mouse postganglionic sympathetic neurons [5]. Voltage-gated Na^+^ channels (VGSCs) consist of pore-forming α-subunits (220–260 kDa) and auxiliary β-subunits (32–36 kDa) [6]. VGSCs regulate the propagation and regeneration of action potential (AP) [7–10]. TNF-α enhances Na^+^ currents in dorsal root ganglion (DRG) neurons [11, 12]. Considering the implication of TNF-α in a variety of pathological processes and diseases of the CNS, we examined the potential effects of TNF-α on voltage-gated Na^+^ currents in primary culture of mouse cortical neurons in the current study.

## Materials and methods

### Cortical neuron culture

The animal study complied with the guidelines for care and use of experimental animals of the Ethics Committee of Shantou University Medical College. Primary culture of cortical neurons was performed using 14-day-old (E14) C57 BL/6 mouse embryos, as described previously [13]. C57 BL/6 mice were purchased from the Shantou University Medical College Experimental Animal Center, Shantou, China. Cerebral cortices (without the hippocampus and duramater) were trypsinized for 2 min with 4 ml of 0.25 % trypsin (Invitrogen) at 37 °C and then interrupted with 0.5 ml of fetal bovine serum (HyClone). Cells were collected by centrifugation for 10 min at 900×*g*. The pellet was re-suspended in minimum essential medium (Invitrogen). Cells (1 × 10^6^) were plated onto 22 × 22 mm poly-D-lysine-pretreated glass cover slips (Sigma) with 2 ml culture medium at 37 °C in 5 % CO_2_/95 % air. Glutamine (2 mM) and B-27 supplement (2 %) were added into the neurobasal medium immediately before use. Half of the culture medium was changed every 3 days. A selective inhibitor of DNA synthesis, arabinosylcytosine C (5 μM), was added to the medium on day 3 for 24 h to minimize glial contamination. All experiments were performed on days 8–12.

### Electrophysiological recording

Electrophysiological recording of the cortical neurons was performed, as previously reported [13].The neurons were re-plated within 24 h before recording in order to minimize space clamp artifacts. Extracellular solution for whole-cell recording of voltage-gated Na^+^ currents consisted of 70 mM NaCl, 70 mM choline-Cl, 5 mM KCl, 1 mM CaCl_2_, 1 mM MgCl_2_, 10 mM HEPES, 4 mM TEA–Cl, 0.1 mM CdCl_2_, and 10 mM glucose (pH adjusted to 7.3 with NaOH). Added to block Ca^2+^ currents was 0.1 mM CdCl_2_. The pipette solution contained 145 mM CsCl, 1 mM MgCl_2_, 5 mM EGTA, 10 mM HEPES, and 5 mM ATP–Na_2_ (pH adjusted to 7.3 with CsOH). Pipettes were pulled using borosilicate glass capillary tubes with a P-97 micropipette puller (Sutter Instrument). Tip resistance was 3–5 MΩ. Voltage clamp recording was performed using an EPC-10 amplifier (HEKA). Series resistance was compensated by 80 to 90 %. Data were digitized at 200 kHz. Neurons were held at −100 mV and depolarized to 100 mV for 20 ms with 5-mV steps and 0.5-Hz frequency. To examine the activation properties of VGSCs, Na^+^ currents were recorded at 0 mV after prepulsing from −100 to 30 mV for 40 ms with 5-mV steps to examine the inactivation properties of VGSCs.

The threshold for APs and neuronal firing rate were recorded in a current clamp mode. The neurons were held at 0 pA, and APs were elicited with depolarizing currents ranging from −50 to 70 pA (120 ms) with increments of 10 pA. The external solution contained 140 mM NaCl, 3 mM KCl, 1 mM MgCl_2_, 1 mM CaCl_2_, 0.1 mM CdCl_2_ and 10 mM HEPES (pH adjusted to 7.3 with NaOH). The pipette solution contained 140 mM KCl, 0.5 mM EGTA, 5 mM Mg-ATP, and 5 mM HEPES (pH adjusted to 7.3 with KOH). Added to block Ca^2+^ currents was 0.1 mM CdCl_2_. All experiments were performed at room temperature (23–25 °C).

### Drug application

Tetrodotoxin (TTX; 300 nM in the bath solution of voltage clamp experiments) was used to verify the TTX sensitivity of the channels. In dose-finding experiments, TNF-α (Sigma) of varying concentration (1, 10, 100, and 1000 pg/ml) was included in the medium for 24 h. For subsequent experiments, cultured neurons were exposed to TNF-α at 100 or 1000 pg/ml in 0.1 % bovine serum albumin (BSA) in saline for 4, 8, 24, or 48 h. Neurons were pretreated with an NF-κB inhibitor (10 μM BAY-11 7082 in 0.1 % dimethylsulfoxide) or a p38 mitogen-activated protein kinases (MAPK) inhibitor (10 μM SB203580 in 0.1 % DMSO) for 0.5 h before and during TNF-α exposure (100 pg/ml 24 h) to determine whether the activation of NF-κB and/or p38 MAPK contributes to the TNF-α-induced enhancement of voltage-gated Na^+^ currents.

### Total RNA extraction and reverse transcription and real-time PCR

The Na_V_1.1, Na_V_1.2, Na_V_1.3, and Na_V_1.6 Na^+^ channels are expressed in cerebrocortex [14]. Potential changes in these channels after TNF-α exposure were examined at the mRNA expression level. TNFR1 expression was examined with real-time quantitative PCR.

Total RNA was extracted using TRIzol reagent (Invitrogen) and reverse-transcribed to cDNA using the following primers: Nav1.1, 5′-CATGTATGCTGCAGTTGATTCCA-3′ (forward) and 5′-AACAGGTTCAGGGTAAAGAAGG-3′ (reverse); Nav1.2, 5′-GCCTTGCTCCTCAGTTCTTTCA-3′ (forward) and 5′-CGGCTATCTGGAGGTTGTTCA-3′ (reverse); Nav1.3, 5′-GGTGTGCCTCATCTTCTGGTTAA-3′ (forward) and 5′-TGCTGCCCGTTGTCATGTTA-3′ (reverse); Nav1.6, 5′-CGTGACACGGTTGCATCCT-3′ (forward) and 5′-ACCGAGTGTGGAACATGCAGTA-3′ (reverse); TNF-RI,5′-CAACGTCCTGACAATGCAGACC-3′ (forward) and 5′-ACGCATGAACTCCTTCCAGCG-3′ (reverse);GAPDH, 5′-ATCAAGAAGGTGGTGAAGCA-3′ (forward) and 5′-AAGGTG GAAGAATGGGAGTTG-3′ (reverse). A total 20-μl reaction volume contained 10-μl SYBR Green PCR Master Mix (Applied Biosystems), 1-μl cDNA template, and 1-μl forward/reverse primers. The templates were amplified using the following protocol: 95 °C for 2 min, 40 cycles of 95 °C for 30 s, and 60 °C for 1 min. TNFR1 mRNA was reverse-transcribed from 1-μg total RNA extract using Expand Reverse Transcriptase (Roche) with the following primers (NM_011609; 228 bp): 5′-TCACCCACAGGGAGTAGGGCA-3′ (forward) and 5′-GCCTGGCGGCGCCGCACGCCG-3′ (reverse). TNFR1 was amplified for 30 cycles at an annealing temperature of 60 °C, followed by a final elongation at 72 °C for 10 min.

### Immunoblot

Membrane preparation, obtained with discontinuous sucrose gradient centrifugation, was used to measure the expression of the pore-forming VGSC α (SCNα) subunit. Briefly, whole-brain lysate in 0.32 M sucrose/5 Mm Tris (pH 7.4) was layered onto 1.2 M sucrose/5 mM Tris (pH 7.4) and then centrifuged at 10,000×*g* for 30 min. The protein fraction at the 0.8/1.2 M sucrose interface was collected, diluted twofold with 0.8 M sucrose/5 mM Tris (pH 7.4), and then centrifuged at 20,000×*g* for 20 min. Pelleted membrane proteins were re-suspended in RIPA buffer containing 25 mM Tris, 150 mM NaCl, 1 mM EDTA, and 2 % Triton X-100 (pH 7.4), and then centrifuged at 20,000×*g* for 20 min. The resulting supernatant was used as membrane preparation for further analysis. Complete Protease Inhibitor (Roche) was included in all solutions. Membrane proteins (100 mg) were fractionated by sodium dodecyl sulfate–polyacrylamide gel electrophoresis, and electro-transferred onto a nitrocellulose membrane (Millipore). The membrane was blocked in 5 % nonfat dry milk and then incubated with a mouse anti-pan Nav antibody (1:200; Alomone) overnight at 4 °C. The membrane was washed with TBS/Tween-20, incubated in horseradish peroxidase-conjugated secondary antibody (goat anti-mouse 1:1,000; Sigma), washed again with TBS/Tween-20, and then visualized using a standard chemiluminescence method.

### Viability experiments

Ten coverslips, each containing at least 100 mouse neurons, were incubated for 24 h in either control medium (*n* = 5 coverslips) or medium containing 100 pg/ml TNF-α (*n* = 5 coverslips), followed by staining with trypan blue (0.2 %) for 5 min. The number of viable cells is expressed as a percentage of the total cell population.

### Statistical analysis

Data are expressed as mean ± SEM. Statistical analysis was performed using Origin software (Origin Lab Corporation, Northampton,) and SPSS 15.0 (SPSS Inc.). The normative Na^+^ current amplitudes, mRNA, and protein expression level were analyzed using two-way ANOVA analysis with treatment and exposure time as independent variables. The *V*_1/2_ and slope (*k*) of activation and inactivation were evaluated using one-way ANOVA. Threshold for AP and firing rate of the two groups were compared using Student’s *t* test. The efficiency of amplification of the target and normalizing products ranged from 0.9~1.1 in real-time PCR. The results were calculated using the 2^−(ΔΔCT)^ method.

## Results

### Concentration-dependent enhancement of Na^+^ currents by TNF-α

**Fig. 1:**
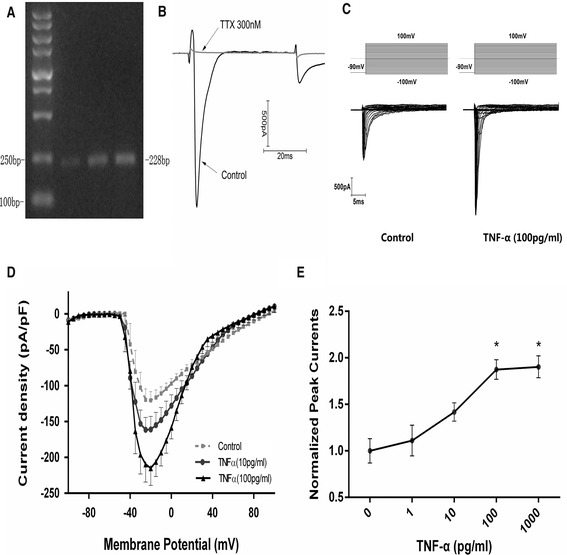
Concentration-dependent effects of tumor necrosis factor-α (TNF-α) on voltage-gated Na^+^ currents in cultured mouse cortical neurons. **a** Expression of TNF-α receptor 1 in mouse cortical neurons as detected by RT-PCR. **b** Original recording curves of whole-cell currents from −80 to 100 mV in control and TTX (300 nM) treated neurons. **c** Typical recording curves of whole-cell Na^+^ currents from −80 to 100 mV in the control and TNF-α-treated (100 pg/ml, 24 h) neurons. **d** Current density–voltage relationship in the control and TNF-α-treated (10 and 100 pg/ml, 24 h) groups. **e** Concentration-dependent effects of TNF-α (1, 10, 100, and 1000 pg/ml) on Na^+^ currents. Data were normalized against the control and expressed as percentage of the control group. *P < 0.05 vs. the control (one-way ANOVA followed by SNK test)

Reverse transcription PCR revealed the presence of TNFR1 mRNA within cortical neurons (Fig. 1a). An inward current with fast activation and inactivation was elicited by depolarization steps from −100 mV of the holding potential and recorded at 200 kHz of the sampling frequency; the currents under were completely blocked with TTX (300 nM; Fig. 1b). TNF-α (1–1000 pg/ml for 24 h) enhanced the voltage-gated Na^+^ currents in a concentration-dependent manner by 11, 41, 80, and 83 % at 1, 10, 100, and 1000 pg/ml, respectively (Fig. 1c–e).The peak currents (at −20 mV), plotted against TNF-α concentration (Fig. 1e), also revealed the action is concentration-dependent.

### Time-dependent enhancement of Na^+^ currents by TNF-α

**Fig. 2:**
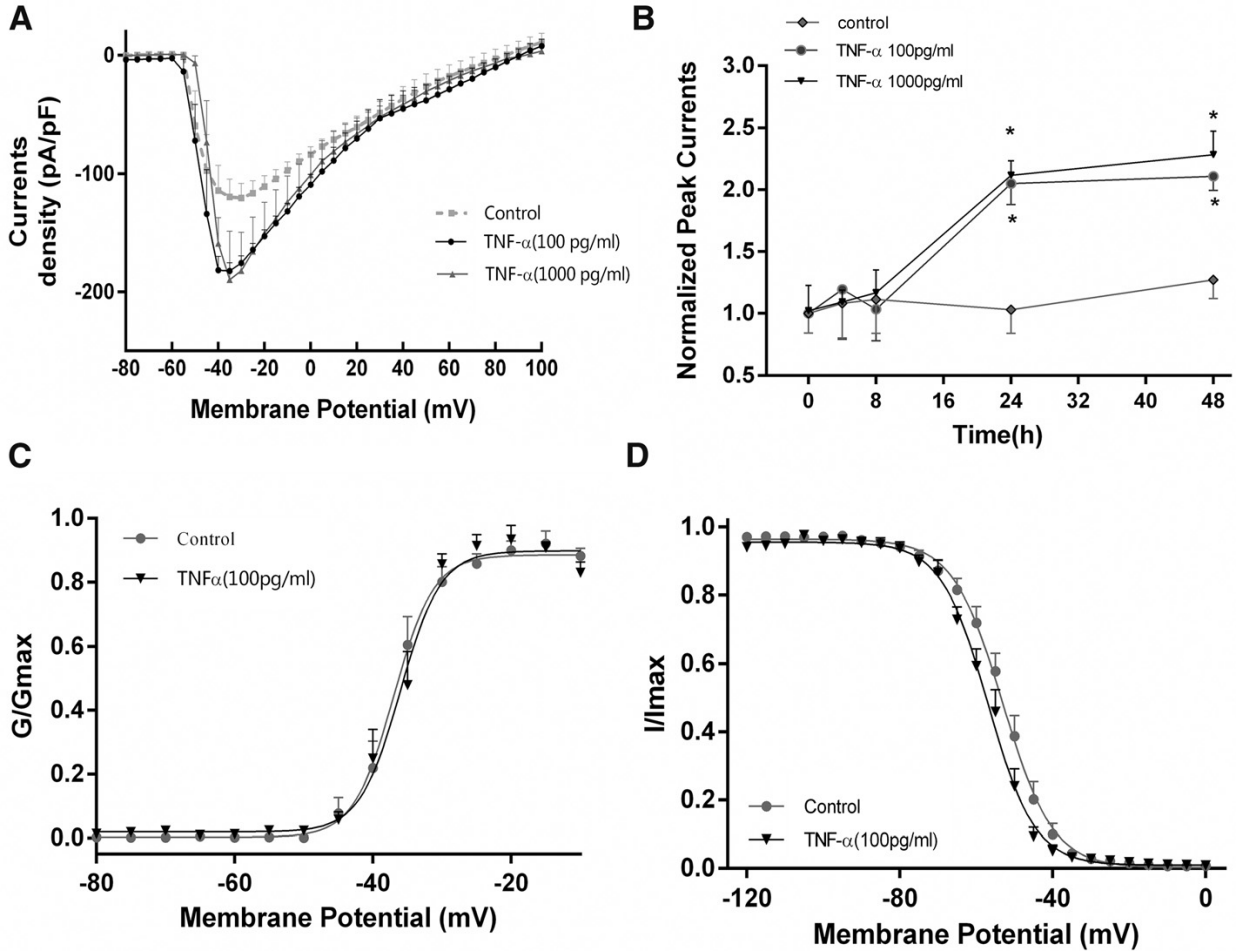
Time-dependent effects of TNF-α on Na^+^ currents and electrophysiological characteristics of Na^+^ currents. **a** Current density–voltage relationship in the group treated with 100 and 1000 pg/ml TNF-α and control group for 24 h. **b** Time dependency of TNF-α (100 and 1000 pg/ml) effects on Na^+^ currents ranging from 0 to 48 h. Current data were normalized by the control and expressed as percentage of the control group. *P < 0.05 as compared with 0 h group, ^*#*^ *P* < 0.05 the 100 or 1000 pg/ml TNF-α-treated group (24 h) vs. the control group (24 h), and 1000 pg/ml TNF-α-treated group (24 h) vs. the control group (48 h) (two-way ANOVA). **c** Voltage-dependent activation curves (Boltzmann equation and fitting parameters) in the 100 pg/ml TNF-α-treated (24 h) and 24 h control groups. **d** Voltage-dependent fast-inactivation curves (Boltzmann equation and fitting parameters) in the 100 pg/ml TNF-α-treated (24 h) and 24 h control groups

The concentration of 100 and 1000 pg/ml was chosen for further experiments to examine the effects of TNF-α. TNF-α increased Na^+^ currents at 24 and 48 h but not after 4 or 8 h exposure (Fig. 2a). Because of the different datasets, the sodium current density for 100 pg/ml in Fig. 2a is different from the one in Fig. 1d. The normative peak current at −20 mV also revealed time-dependent increase of the TNF-α action (Fig. 2b).

### Effects of TNF-α on the activation and fast-inactivation of Na^+^ channels

The conductance–voltage curve, fitted using the Boltzmann equation, is shown in Fig. 2c. Fast-inactivation, reflected as the peak currents at 0 mV test voltage normalized using the maximal current, is shown in Fig. 2d. Normative currents were plotted to the prepulse voltage, and the current–voltage curves were also fitted using the Boltzmann equation (Fig. 2d). The voltage dependence (*V*_1/2_) and slope for both activation and steady-state inactivation are shown in Table 1.

**Table 1:** The effect of TNF-α (100 pg/ml 24 h) on Na^+^ channel kinetics

	Activation		Inactivation	
	Control (*n* = 10)	TNF-α (*n* = 17)	Control (*n* = 15)	TNF-α (*n* = 19)
*V*_1/2_ (mV)	−37.01 ± 0.46	36.07 ± 0.48	−53.07 ± 0.43	56.95 ± 0.39^*^
Slope factor	2.907 ± 0.39	2.929 ± 0.42	6.528 ± 0.38	6.117 ± 0.33

*V*_1/2_ voltage of half-maximal activation or inactivation*, k* slope, TNF-α (100 pg/ml, 24 h)

^*^ *P <* 0.001 vs. the control

TNF-α hyperpolarized shift *V*_1/2_ of inactivation from −53.07 ± 0.43 (*n* = 15) to −56.95 ± 0.39 mV (*n* = 19), without affecting *k* (Table 1 and Fig. 2d). Channel activation was not affected by TNF-α (Fig. 2c). TNF-α exposure did not affect neuronal viability (67.9 ± 1.8 % vs. 69.3 ± 2.1 %; *P* = 0.69, Mann–Whitney test).

**Fig. 3:**
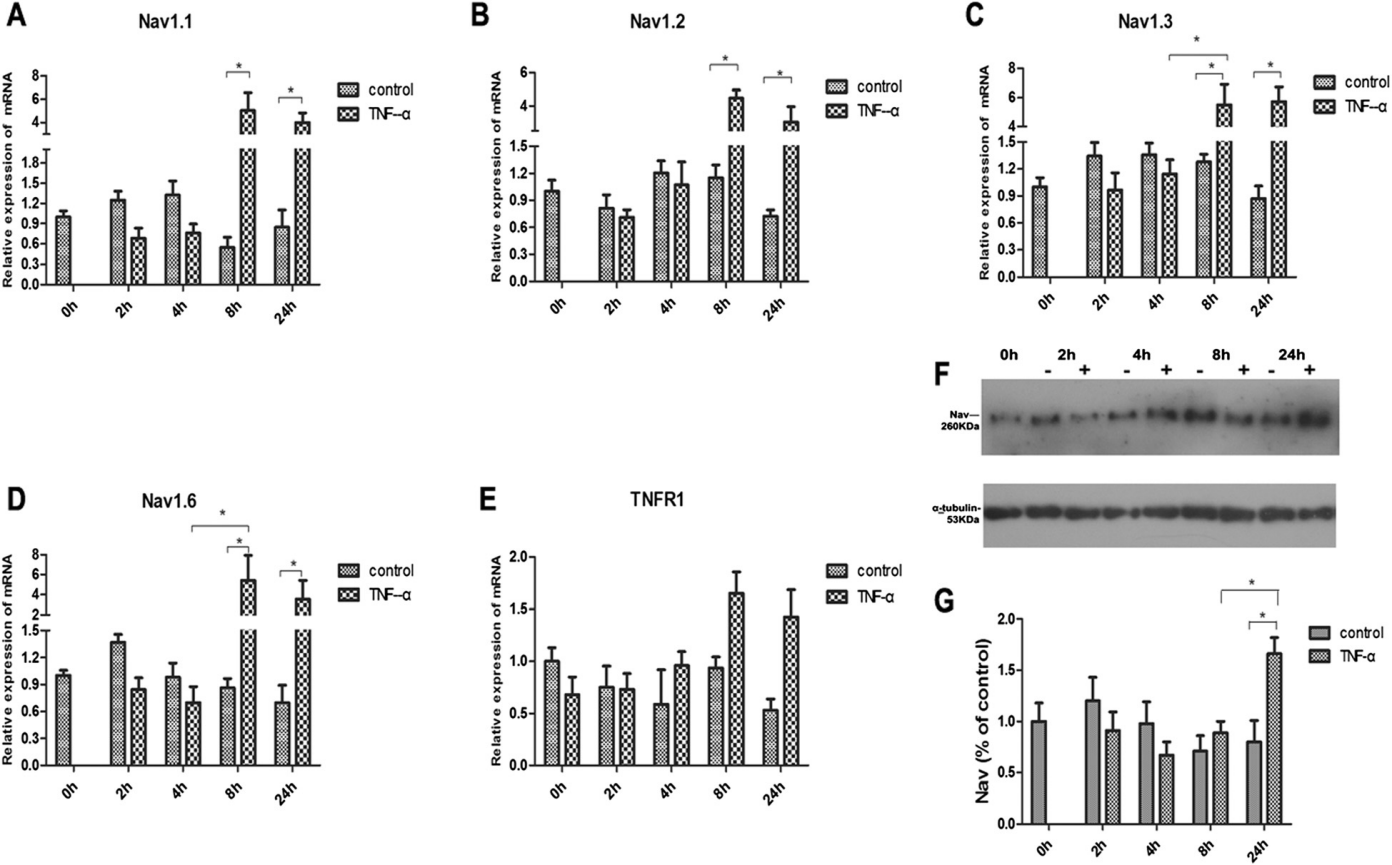
Effects of TNF-α on the expression of TNFR1 mRNA and VGSCs. Normative expression level of Nav1.1 (**a**), Nav1.2 (**b**), Nav1.3 (**c**), Nav1.6 (**d**), and TNF-R1 (**e**) mRNA. The value of each group was obtained from three separate experiments done in duplicates. *P < 0.05 vs. the relevant control (two-way ANOVA). **f** Nav channels protein expression in cortical neurons were detected with western blot “−”: treated with 0.1 % BSA in PBS buffer, “+”: treated with TNF-α (100 pg/ml) in PBS buffer. **g** The value of each group was obtained from three separate experiments. *P < 0.05 (two-way ANOVA)

TNF-α treatment (100 and 1000 pg/ml) markedly increased the level of mRNAs for Nav1.1, Nav1.2, Nav1.3, and Nav1.6 at 8 and 24 h but not upon shorter incubation (Fig. 3a–d). TNF-α produced a tendency for increasing TNFR1 mRNA at 8 and 24 h, but the results were not statistically significant at 24 h (Fig. 3e). GAPDH was used in the present study as the reference gene because of its stability under a wide range of experimental treatments. All these data come from three separate experiments.

Consistent with the patch-clamp experiments, immunoblot of Nav channels in the plasma membrane of VGSC (Fig. 3f) showed significantly increased number of Nav channels (by 65.88 %) after TNF-α treatment (100 pg/ml) for 24 h (*P* < 0.05) (Fig. 3g).

### BAY 11–7082 and SB 203580 inhibited the effects of TNF-α on voltage-gated Na^+^ currents

**Fig. 4:**
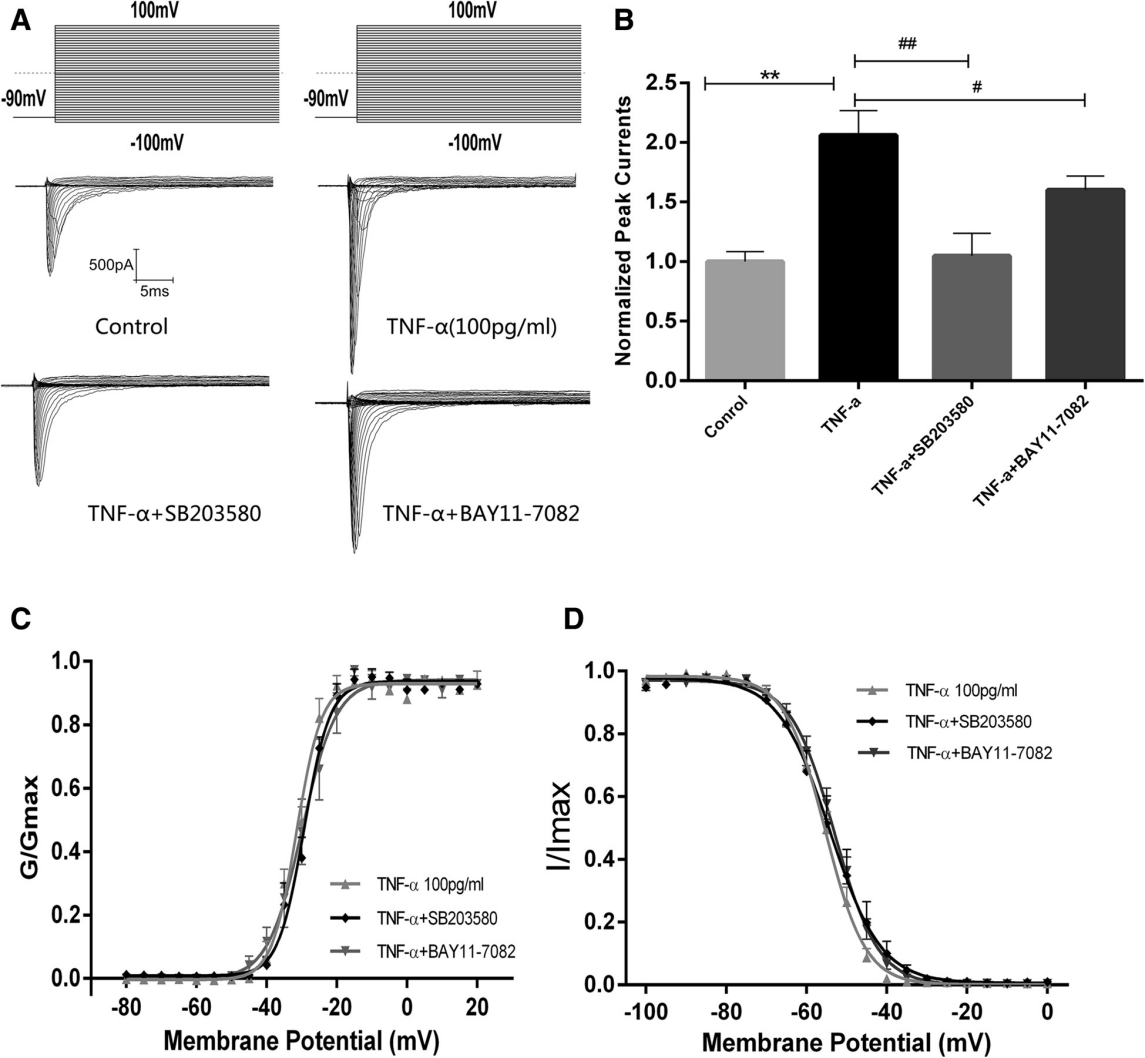
Inhibition of NF-κB and p38 MAPK decreased the TNF-α augmentation of Na^+^ currents. **a** Typical recording curves of whole-cell Na^+^ currents from −80 to 100 mV in the control and TNF-α (100 ng/ml, 24 h), TNF-α + BAY 11-7082(1 uM), and TNF-α + SB203580 (1 uM)groups. **b** Normalized peak current–voltage relationship in the control and TNF-α (100 pg/ml, 24 h), TNF-α + BAY 11-7082(1 uM), and TNF-α + SB203580 (1 uM) groups. *P < 0.05 vs. the 24 h control group; ^*#*^ *P < 0.05* vs. TNF-α (100 pg/ml, 24 h) group (one-way ANOVA followed by SNK). **c** Voltage-dependent activation curves (Boltzmann equation and fitting parameters) in the TNF-α (100 pg/ml, 24 h), TNF-α + BAY 11-7082, and TNF-α + SB203580 groups. **d** Voltage-dependent inactivation curves (Boltzmann equation and fitting parameters) in the TNF-α (100 pg/ml, 24 h), TNF-α + BAY 11-7082, and TNF-α + SB203580 groups

Previous studies have reported that TNFR1 activation mediates the effects of soluble TNF-α on various cell processes, including gene transcription, via downstream intracellular signaling pathways by activating the NF-ĸB signaling complex [15] and/or p38 MAPK [16]. In our experiments, pretreatment with either BAY 11–7082 or SB203580 for 30 min before TNF-α treatment attenuated the effects of TNF-α (by 98 and 37 %, respectively; Fig. 4a, b). BAY 11–7082 also induced a depolarization shift of *V*_1/2_ in steady-state fast-inactivation from −55.33 ± 0.31 (*n* = 9) to −53.04 ± 0.34 mV (*n* = 8, *p* < 0.001, as compared with TNF-α) (Fig. 4d and Table 2) but not activation (Fig. 4c). SB203580 did not affect the gating properties (either *V*_1/2_ or *k*) of either activation or steady-state inactivation (Fig. 4c, d).

**Table 2:** Effects of SB203580 and BAY11-7082 on Na^+^ channel kinetics in neurons treated by TNF-α

	Activation			Inactivation		
	TNF-α (*n* = 7)	TNF-α + SB203580 (*n* = 12)	TNF-α + BAY11-7082 (*n* = 15)	TNF-α (*n* = 9)	TNF-α + SB203580 (*n* = 9)	TNF-α + BAY11-7082 (*n* = 8)
*V*_1/2_ (mV)	−31.47 ± 0.38	−29.32 ± 0.36	−29.9 ± 0.62	−55.33 ± 0.31	−54.21 ± 0.61	53.04 ± 0.34^*^
Slope factor	3.553 ± 0.31	3.681 ± 0.31	4.867 ± 0.54	5.003 ± 0.27	6.380 ± 0.54	5.453 ± 0.30

*V*_1/2_ voltage of half-maximal activation or inactivation*, k* slope, TNF-α (100 pg/ml, 24 h)

^*^ *P* < 0.001 vs. TNF-α (100 pg/ml, 24 h) group (one-way ANOVA followed by SNK)

### Effects of TNF-α on the amplitude and threshold of AP

**Fig. 5:**
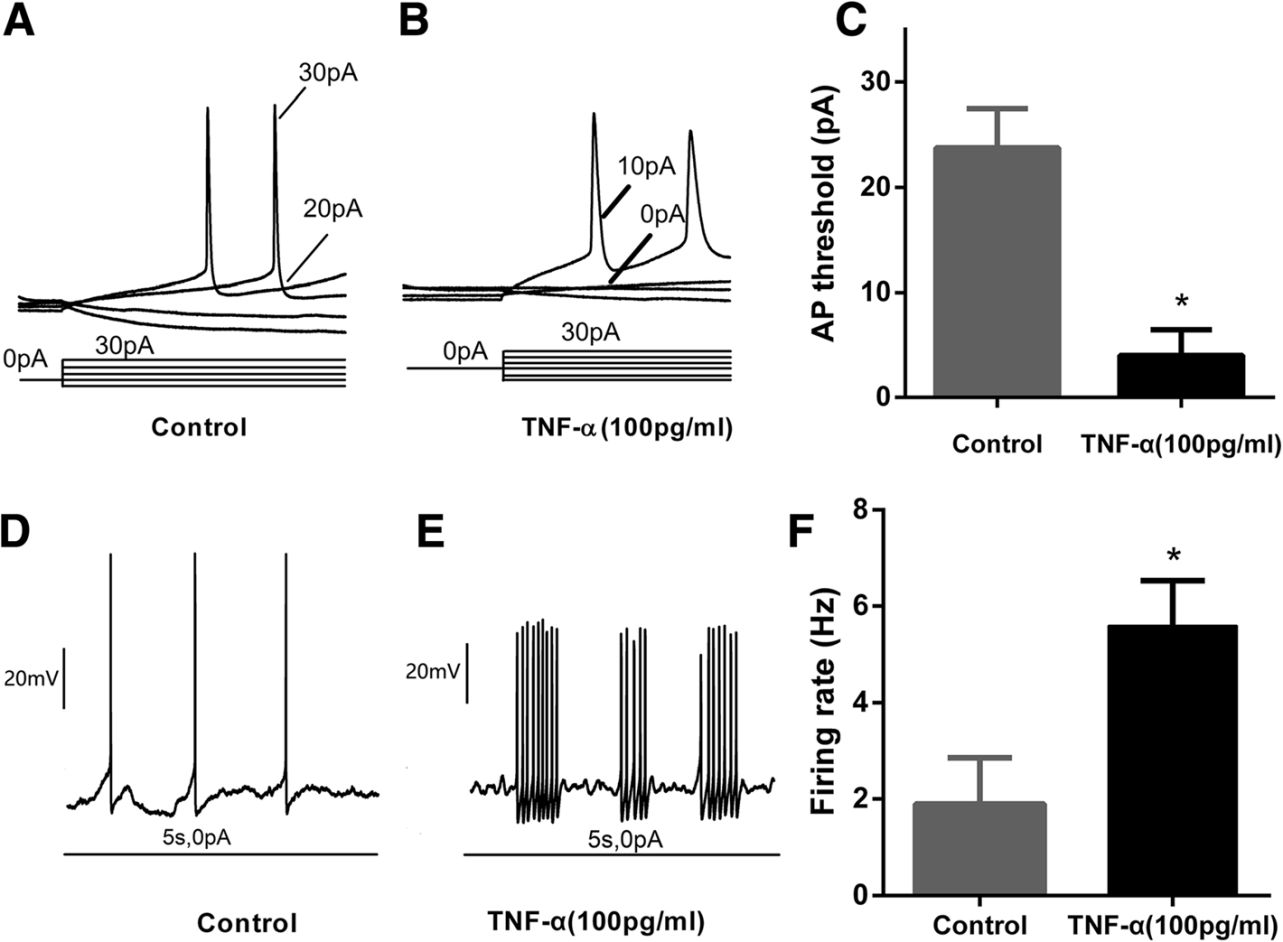
Effects of TNF-α on spike. **a**, **b** Traces showing representative recordings of action potentials. Threshold of action potential was significantly lower in the TNF-α group than in the control group. **c** Histogram showing the action potential threshold in the control and TNF-α groups, (*P < 0.05, *n* = 10 in each group (Student’s t-test). **d** and **e** Representative recordings of firing rate of action potential in response to current injection (500 pA, 1 s) in the control and TNF-α (100 pg/ml for 24 h) groups. **f** Histogram showing the firing rate in the control and TNF-α (100 pg/ml for 24 h) groups. *P < 0.05, Student’s *t*-test (*n* = 8 in each group)

The threshold of the spike was significantly lowered by TNF-α (100 pg/ml, 24 h) (Fig. 5a–c). The firing rate was increased by TNF-α (100 pg/ml, 24 h) (Fig. 5d–f). These results, consistent with the data from voltage clamp recording, strongly indicated that TNF-α can increase the excitability of mouse cortical neurons.

## Discussion

Long-term treatment (12 h) with TNF-α enhances the currents of VGSCs in DRG neurons following motor nerve injury and increases the expression of Nav1.3, Nav1.6 (TTX-S) and Nav1.8 (TTX-R) [17–19]. In the present study, we found that TNF-α could enhance voltage-gated Na^+^ currents of neurons in the CNS in a time- and concentration-dependent manner. We also showed that TNF-α accelerates the inactivation of Na^+^ channels, and the effect could be attenuated by BAY11-7082, a NF-ĸB inhibitor. The increased VGSC function was associated with increased expression of VGSCs at both the mRNA and protein levels. Effects of TNF-α on Na^+^ currents were apparently dependent on NF-κB and p38 MAPK signaling pathways. Together with previous reports, these results indicated an important role of the pro-inflammatory cytokine of TNF-α in neuron excitability.

Na^+^ currents increased only after 24 h, but not earlier, after TNF-α exposure in our experiments. In a preliminary set of acute experiments (5-min exposure), TNF-α increased Na^+^ channels only at 10,000 pg/ml but not the concentrations (100 and 1000 pg/ml) used in the experiments (hours of incubation, as described earlier). While previous work found that bath application of a mouse recombinant TNF-α (100 ng/ml) could rapidly enhance peak TTX-R Na^+^ currents (within 3–5 min) in DRG neurons in a concentration-dependent manner at 20–100 ng/ml [17]. The obvious differences in the required concentration of TNF-α may be dependent on the subtypes of VGSCs and neurons.

In our results (Fig. 2c), TNF-α appears to increase persistent sodium currents, which likely resulted from enhanced expression of Nav1.6 channels. Nav1.6 channel is known to be capable of producing persistent sodium current which can affect firing threshold and sustained firing [20, 21]. While the persistent Na^+^ current is typically a small fraction of the peak current in neurons, it plays a critical role in firing regulation. Persistent Na^+^ currents cause the activation of a subthreshold plateau potential and helped initiate each spike and maintain depolarization of membrane potential near threshold permitting firing. Therefore, it is possible that TNF-α elevates neuronal excitability via enhanced persistent Na^+^ currents.

Among the multitudinous cytokines produced by immune cells (microglia) in the brain, TNF-α is a central mediator of inflammation, immune response, and antiviral defense [22]. Both neurotoxic and neuroprotective effects have been attributed to TNF-α in the CNS, possibly due to activation of different TNF-α receptors [16, 23]. TNFR1 is constitutively expressed on variety of cell types, including neurons in brain [24], whereas TNFR2 is highly regulated and is typically found on microglia and endothelial cells [25]. Consistent with previous studies, we confirmed the presence of TNFR1 but not TNFR2 in cortex neurons by real-time PCR and immunofluorescence (data not shown).

Soluble TNF-α exerts its effects mainly through TNFR1 [15], and TNFR1 signaling is functionally important for homeostatic process in CNS [26]. TNF-α can potentiate excitotoxicity by potentiating glutamate excitotoxicity and inhibiting glial glutamate transporters through TNFR1 signal on astrocytes [27, 28]. In the present study, TNF-α increased Na^+^ current density and generation of Aps, suggesting TNFR1 likely directly potentiates excitotoxicity by enhancing functional voltage-gated sodium channels in cortex neurons.

The activation of many pivotal signal pathways (including p38 MAPK pathway and NF-κB pathway) by TNF-α in the CNS is mediated by TNFR1 [2]. The p38 MAPK signal pathway seemingly has a multiple and contradictory roles in Na^+^ currents in neurons. Earlier studies document that TNF- α enhanced the currents of VGSCs in DRG neurons and increases the expression of Nav1.3, Nav1.6, and Nav1.8 after 12-h treatment [17–19]. Similarly, in this study, a chronic exposure (24 h) of neurons to TNF-α induced an apparent increase of Na^+^ currents by increasing VGSC mRNA and protein levels. In our experiments, potentiation of TNF-α was blocked by the p38 MAPK inhibitor SB203580, possibly by inhibiting constitutive p38 MAPK activation as reported earlier in normal neurons [29]. In contrast, acute exposure (within 1 h) of TNF-α enhances TTX-R (Nav1.8) Na^+^ currents by p38-mediated modulation in sensory neurons and DRG neurons [17, 19], due to p38 MAPK-mediated phosphorylation of serine residues within L1 of the channel. Wittmack and Gasser independently found that acute activation of p38 leads to a significant reduction in the peak Nav1.6 current amplitude in the neuronal ND7/23 cell line and native hippocampal neurons by phosphorylation of specific polypeptide motif within L1 of Nav1.6 [29, 30]. For both Na^+^ current increase in TTX-R VGSCs and reduction in TTX-S VGSCs, the underlying mechanism is based on p38 MAPK-mediated phosphorylation of the channels. Such a mechanism is apparently distinct from what happens upon prolonged exposure to TNF-α: p38 MAPK-mediated Na^+^ current enhancement by increasing the channel synthesis. Together with these previous results, the regulation of VGSC by p38 MAPK that we report in this study demonstrates that the mechanism of p38 MAPK-mediated voltage gated Na^+^ channels regulation dependent on exposure duration and the type of the neurons.

Earlier study confirmed that the phosphorylated forms of IKK-1, IKK-2, and IκB-α are concentrated within the axon initial segment (AIS) and the node of Ranvier [31]. These axon potential generating regions are rich in membrane-embedded voltage-gated sodium channels. In the present study, chronic exposure of TNF-α induced NF-κB activation associating with enhancive Na^+^ currents by increasing voltage-gated sodium channels expression. The important roles of NF-κB in modulation of voltage-dependent calcium channels by TNF-α have also been reported in cultured rat hippocampal neurons [32]. Furthermore, inhibition of NF-κB attenuated the shift of *V*_1/2_ of inactivation induced by TNF-α, investigating that NF-κB is involve with VGSC dynamic property. In contrast, both the current study and previous reports [29, 30] showed that the p38 MAPK inhibitor SB203580 does not affect the activation and inactivation.

In summary, we showed that TNF-α enhances the activity of VGSCs in cultured mouse cortical neurons in a time- and concentration-dependent manner. This enhancement is associated with the alteration of channel properties as well as upregulation of VGSCs. The effects were likely dependent on the TNFR1-mediated NF-κB and p38 MAPK signaling pathways. Our findings encourage further studies to examine the effect of TNF-α in the brain in the context of excitotoxicity induced by inflammatory and CNS diseases.

## References

[1] Montgomery SL, Bowers WJ (2012). Tumor necrosis factor-alpha and the roles it plays in homeostatic and degenerative processes within the central nervous system. J Neuroimmune Pharmacol..

[2] Lee TH, Huang Q, Oikemus S, Shank J, Ventura JJ, Cusson N (2003). The death domain kinase RIP1 is essential for tumor necrosis factor alpha signaling to p38 mitogen-activated protein kinase. Mol Cell Biol..

[3] Fontaine V, Mohand-Said S, Hanoteau N, Fuchs C, Pfizenmaier K, Eisel U (2002). Neurodegenerative and neuroprotective effects of tumor necrosis factor (TNF) in retinal ischemia: opposite roles of TNF receptor 1 and TNF receptor 2. J Neurosci..

[4] Liu BG, Dobretsov M, Stimers JR, Zhang JM (2008). Tumor necrosis factor-α suppresses activation of sustained potassium currents in rat small diameter sensory neurons. Open Pain J..

[5] Motagally MA, Lukewich MK, Chisholm SP, Neshat S, Lomax AE (2009). Tumour necrosis factor alpha activates nuclear factor kappaB signalling to reduce N-type voltage-gated Ca^2+^ current in postganglionic sympathetic neurons. J Physiol..

[6] Catterall WA (2000). From ionic currents to molecular mechanisms: the structure and function of voltage-gated sodium channels. Neuron..

[7] Hodgkin AL, Huxley AF (1952). A quantitative description of membrane current and its application to induction and excitation in nerve. J Physiol..

[8] Hille B (2001). Ion channels of excitable membranes.

[9] Goldfarb M, Schoorlemmer J, Williams A, Diwakar S, Wang Q, Huang X (2007). Fibroblast growth factor homologous factors control neuronal excitability through modulation of voltage-gated sodium channels. Neuron..

[10] Rush AM, Cummins TR (2007). Painful research: identification of a small-molecule inhibitor that selectively targets Nav1.8 sodium channels. Mol Interv.

[11] He XH, Zang Y, Chen X, Pang RP, Xu JT, Zhou X (2010). TNF-α contributes to up-regulation of Nav1.3 and Nav1.8 in DRG neurons following motor fiber injury. Pain.

[12] Chen X, Pang RP, Shen KF, Zimmermann M, Xin WJ, Li YY (2011). TNF-α enhances the currents of voltage gated sodium channels in uninjured dorsal root ganglion neurons following motor nerve injury. Exp Neurol..

[13] Chen W, Zhu F, Guo J, Sheng J, Li W, Zhao X (2014). Chronic haloperidol increases voltage-gated Na + currents in mouse cortical neurons. Biochem Biophys Res Commun.

[14] Catterall WA, Goldin AL, Waxman SG (2005). International union of pharmacology. XLVII. Nomenclature and structure-function relationships of voltage-gated sodium channels. Pharmacol Rev.

[15] Grell M, Douni E, Wajant H, Lohden M, Clauss M, Maxeiner B (1995). The transmembrane form of tumor necrosis factor is the prime activating ligand of the 80 kDa tumor necrosis factor receptor. Cell..

[16] Baud V, Karin M (2001). Signal transduction by tumor necrosis factor and its relatives. Trends Cell Biol..

[17] Jin X, Gereau RW (2006). Acute p38-mediated modulation of tetrodotoxin-resistant sodium channels in mouse sensory neurons by tumor necrosis factorα. J Neurosci..

[18] Shen KF, Zhu HQ, Wei XH, Wang J, Li YY, Pang RP (2013). Interleukin-10 down-regulates voltage gated sodium channels in rat dorsal root ganglion neurons. Exp Neurol..

[19] Hudmon A, Choi JS, Tyrrell L, Black JA, Rush AM, Waxman SG (2008). Phosphorylation of sodium channel Na(v)1. 8 by p38 mitogen-activated protein kinase increases current density in dorsal root ganglion neurons. J Neurosci.

[20] Yuan C, Frank HY, Sharp EM, Beacham D, Todd S, William AC (2008). Functional properties and differential neuromodulation of Na(v)1.6 channels. Mol Cell Neurosci.

[21] Yunru L, Monica AG, David JB (2004). Role of persistent sodium and calcium currents in motoneuron firing and spasticity in chronic spinal rats. J Neurophysiol..

[22] Kraft AD, McPherson CA, Harry GJ (2009). Heterogeneity of microglia and TNF signaling as determinants for neuronal death or survival. Neurotoxicology..

[23] MacEwan DJ (2002). TNF ligands and receptors-a matter of life and death. Br J Pharmacol..

[24] Bette M, Kaut O, Schäfer MK, Weihe E (2003). Constitutive expression of p55TNFR mRNA and mitogen-specific up-regulation of TNF alpha and p75TNFR mRNA in mouse brain. J Comp Neurol..

[25] Wajant H, Pfizenmaier K, Scheurich P (2003). Tumor necrosis factor signaling. Cell Death Differ..

[26] McCoy MK, Tansey MG (2008). TNF signaling inhibition in the CNS: implications for normal brain function and neurodegenerative disease. J Neuroinflammation..

[27] Zou JY, Crews FT (2005). TNF alpha potentiates glutamate neurotoxicity by inhibiting glutamate uptake in organotypic brain slice cultures: neuroprotection by NF kappa B inhibition. Brain Res..

[28] Choi DW (1988). Glutamate neurotoxicity and diseases of the nervous system. Neuron..

[29] Gasser A, Cheng X, Gilmore ES, Tyrrell L, Stephen G (2010). Two Nedd4-binding motifs underlie modulation of sodium channel Nav1.6 by p38 MAPK. J Biol Chem.

[30] Wittmack EK, Rush AM, Hudmon A, Waxman SG, Dib-Hajj SD (2005). Voltage-gated sodium channel Nav1.6 is modulated by p38 mitogen-activated protein kinase. J Neurosci.

[31] Schultz C, Konig HG, Del Turco D, Politi C, Eckert GP, Ghebremedhin E (2006). Coincident enrichment of phosphorylated I kB a, activated IKK, and phosphorylated p65 in the axon initial segment of neurons. Mol Cell Neurosci..

[32] Furukawa K, Mattson MP (1998). The transcription factor NF-κB mediates increases in calcium currents and decreases in NMDA- and AMPA/kainate-induced currents induced by tumor necrosis factor-α in hippocampal neurons. J Neurochem..

